# Default Mode Network Resting State Connectivity Derived From Task‐Based fMRI: A Validation Study in People With Epilepsy

**DOI:** 10.1111/jon.70129

**Published:** 2026-02-21

**Authors:** Lea Wemheuer, Anna Doll, Martin Wegrzyn, Markus Mertens, Johanna Kissler, Christian G. Bien, Friedrich G. Woermann, Philip Grewe

**Affiliations:** ^1^ Medical School OWL, Clinical Neuropsychology and Epilepsy Research Bielefeld University Bielefeld Germany; ^2^ Medical School OWL, Bethel Epilepsy Center, Department of Epileptology, Krankenhaus Mara Bielefeld University Bielefeld Germany; ^3^ Department of Psychology, Affective Neuropsychology Bielefeld University Bielefeld Germany; ^4^ Bethel Epilepsy Center, MRI‐Department, Krankenhaus Mara Society for Epilepsy Research Bielefeld Germany; ^5^ Center for Cognitive Interaction Technology (CITEC) Bielefeld University Bielefeld Germany; ^6^ Medical School OWL, Medical Psychology Bielefeld University Bielefeld Germany

**Keywords:** default mode network, epilepsy, functional connectivity, functional neuroimaging, resting state, task‐based fMRI

## Abstract

**Background and Purpose:**

Resting state functional connectivity can be measured using resting state functional MRI (fMRI), but also task‐dependent fMRI in blocked designs. The latter has been demonstrated in healthy participants but not yet validated in clinical cohorts. Since functional connectivity of resting state networks (e.g., default mode network [DMN] and somatomotor network [SMN]) is altered in people with epilepsy, and the impact of the disease on the quality of the intermittent resting state data is unclear, we aimed to validate the method using a clinical fMRI in people with epilepsy.

**Methods:**

We compared functional connectivity derived from a standard resting state with rest periods of a clinical language fMRI (intermittent resting state) of 92 people with focal epilepsy. Both methods were analyzed across different aspects of functional connectivity: topography, within‐network connectivity, and group‐level comparisons. Therefore, we conducted independent component analyses (ICAs), similarity‐, regions of interest (ROI)‐to‐ROI‐, and second‐level seed‐based analyses.

**Results:**

Results indicated similar ICA‐derived topography of DMN and SMN from both methods. Within‐network connectivity also yielded comparable results. Seed‐based analyses of left and right hippocampal connectivity in people with left and right temporal lobe epilepsy also revealed analogous results, with minor restrictions in right hippocampal connectivity.

**Conclusion:**

The intermittent resting state method produces highly similar results to a standard resting state method in people with epilepsy across different aspects of functional connectivity. It is, therefore, an efficient approach to gain insights into functional connectivity networks in a clinical cohort without performing an additional resting state fMRI.

## 1 Introduction

Even when the brain is not performing an explicit cognitive task, it is never fully at rest and always active in some way [1]. Resting state functional connectivity reflects this intrinsic (co)activation of the brain during a baseline state of relaxed wakefulness [2, 3]. Functional connectivity describes the temporal association of different brain regions showing correlated activation patterns [1, 4].

When multiple brain regions are functionally connected and coactivated, they form a brain network [3]. Networks that do not specifically reflect task effects and occur in the absence of an explicit task—reflecting a baseline state of the brain—are called resting state networks [5, 6] and can represent units of cognitive functions [3, 6]. One of the most commonly known resting state networks is the default mode network (DMN) [6, 7, 8, 9]. It describes a network of increased intrinsic brain activation in the absence of explicit sensory or cognitive stimulation that shows decreased activation during most cognitive tasks [2, 8, 10, 11]. The DMN mainly comprises regions within the ventral and dorsal medial prefrontal cortex (MPFC), the posterior cingulate cortex (PCC), precuneus (PCu), and the lateral parietal cortex [9, 10, 12].

For the identification of a resting state network—like the DMN—functional magnetic resonance imaging (fMRI) can be used. This usually involves a resting state fMRI scan lasting several minutes, during which participants are not performing an active task and are instructed to think of nothing in particular [2, 3]. A more recent approach is to derive resting state functional connectivity from task‐dependent fMRI measures [13, 14]. For this purpose, a blocked task design with rather short rest periods between task blocks (lasting several seconds) can be used [13]. This approach yields quantitatively and qualitatively similar functional connectivity as the standard resting state measurement [13]. Despite its application in healthy subjects or those with neurological disorders such as multiple sclerosis, to the best of our knowledge, this method has not yet been applied in people with epilepsy [13, 14, 15, 16, 17].

People with epilepsy show alterations in functional connectivity compared to healthy individuals [18, 19, 20, 21]. The analysis of resting state networks is, therefore, a valuable approach for clinical use to gain knowledge about the disease and associated deficits of cognitive functions. Resting state fMRI can be used for a variety of clinical purposes: In addition to language lateralization [22, 23], it can be used for localization of epileptic seizure activation [24, 25], prediction of postsurgical (cognitive) outcome—such as memory and language functions [26, 27]—and seizure recurrence and susceptibility [28, 29]. The DMN is a particularly relevant resting state network for clinical application in people with epilepsy [26, 30, 31, 32]. In addition to changes in DMN connectivity, alterations in the somatomotor network (SMN) have also been reported in people with epilepsy [33, 34]. In presurgical diagnostics, task‐fMRI is the standard assessment for language lateralization of people with epilepsy [35]. This language task‐fMRI in a blocked design consists of alternating task and rest blocks [36]. Based on the methodology by Fair et al. [13], one might extend the use of these clinically frequently applied data beyond their original purpose, allowing an economical approach without additional resting state scans to obtain similar information.

The method by Fair et al. [13] had previously been applied in people with neurological diseases [15, 16], but the validation of the method was conducted in healthy individuals [13, 14, 17], thus, the impact of the disease on the quality of the data remains unclear. In general, there is a lack of research in the field of network analyses in various forms of epilepsy, calling for innovative and easily accessible approaches to gain novel scientific insights into the brain networks of people with epilepsy.

The current study tries to validate the method introduced by Fair et al. [13] using rest periods from a blocked task‐dependent language lateralization fMRI task (intermittent resting state) in people with epilepsy. It examines the applicability of the intermittent resting state method across different aspects of functional connectivity: topography, within‐network connectivity, and second‐level group comparisons. We hypothesize that compared to the standard resting state fMRI, the intermittent resting state can be used similarly to derive a DMN in people with epilepsy, and both methods do not significantly differ in their topography of the DMN, within‐network connectivity, and second‐level group comparison results. To broaden applicability and validation of the method in people with epilepsy, in addition to the DMN, the SMN was analyzed.

## 2 Methods

**FIGURE 1:**
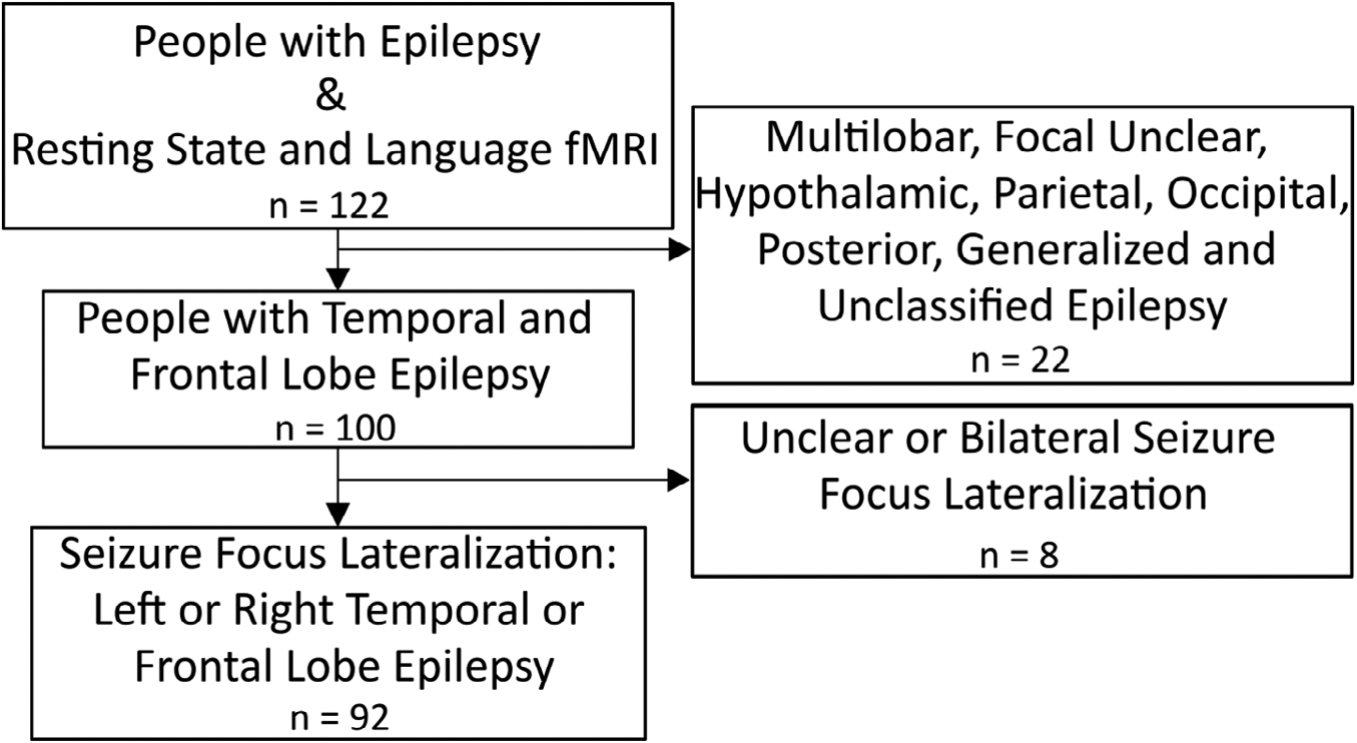
Inclusion and exclusion criteria. Abbreviation: *n*, number.

The sample consisted of 122 people with epilepsy who underwent presurgical assessment at Mara hospital (Medical School OWL, Bielefeld University) at the Bethel Epilepsy Center. In these people, both a language fMRI and a resting state fMRI were conducted. A subset of the sample has previously been part of studies by Doll et al. [32, 37, 38]. Exclusion criteria can be found in Figure 1. Ninety‐two (70 with temporal‐, 22 with frontal‐lobe epilepsy) patients remained for subsequent analysis. We chose this sample as it presents the most typical types of epilepsies that occur in presurgical diagnostics. We, therefore, analyze a rather homogeneous sample, and variance should primarily be accounted for by the two methods. Table 1 displays further demographic information of the sample.

**TABLE 1:** Clinical and demographic sample characteristics.

	TLE (*N* = 70)	FLE (*N* = 22)
	Frequency M (SD)	Frequency M (SD)
Sex (f/m)	32/38	10/12
Age (years)	37.29 (12.64)	32.14 (12.59)
Age at onset (years)	22.25 (10.91)	15.27 (15.10)
Laterality epilepsy (l/r)	41/29	8/14
Laterality language (l/b/i)	62/6/2	19/3/−
Surgery (y/n)	46/24	16/6
ASM	1.77 (0.62)	2 (0.816)
Handedness (l/r)	2/68	2/20

Abbreviations: ASM, number of antiseizure medication; b, bilateral; f, female; FLE, frontal lobe epilepsy; i, inconclusive; l, left; m, male; M, mean; n, no; *N*, number; r, right; SD, standard deviation; TLE, temporal lobe epilepsy; y, yes.

### 2.1 MRI Data Acquisition

MRI data were collected on a 3T Siemens Magnetom Verio MRI scanner. High‐resolution T1‐weighted structural data were acquired using a 32‐channel head coil with 192 sagittal slices with a slice thickness of 0.8 mm, 0.75 × 0.75 mm in‐plane resolution, a field of view of 15.36 × 24 × 24 cm, repetition time of 1.9 s, and echo time of 2.5 ms. For the language fMRI, a 12‐channel head coil was used, acquiring 21 axial slices per volume. Slice thickness was 5 mm and in‐plane resolution 3×3 mm. A repetition time of 3 s was used with an effective acquisition time of 1.8 s and an echo time of 50 ms. The block‐design task used for the language fMRI is described in detail in Woermann et al. [36] and consists of each ten 30‐s blocks of task (language, i.e., word fluency task) alternated with 30‐s rest blocks. In the word fluency task, participants are asked to covertly generate words from a specific semantic category (e.g., animals or names), which are verbally cued; the end of each task block was indicated with an auditory cue (i.e., “Stop”). Task‐free resting state fMRI was collected using a 32‐channel head coil with 34 slices of 4 mm thickness, in‐plane resolution of 2.4×2.4 mm, and a field view of 14×19.2×19.2 cm. Repetition time was 3 s with an echo time of 33 ms. Participants were instructed to keep their eyes open and think of nothing in particular for the duration of the 8.5 min scan.

### 2.2 Data Preprocessing

fMRIPrep version 1.4.1rc4 [39] (Stanford Center for Reproducible Neuroscience, Stanford, CA) and Nilearn [40] version 0.10.2 on Python 3.9.17 were used for preprocessing of the MRI data. Susceptibility distortion was corrected using the fieldmap‐less approach of fMRIPrep. Images were slice‐time corrected and coregistered to MNI152NLin2009cAsym standard space. Additionally, a set of physiological regressors were extracted to allow for component‐based noise correction (CompCor) [41], including motion parameters. For quality check, plots of the six motion parameters (i.e., rotation along the x, y, and z axes, as well as translation in the x, y, and z planes) averaged over all people are displayed (see Figure 2A). Both methods show fluctuations over time, with the continuous resting state measurement displaying an increasing drift in the course of the measurement. This could hint toward the influence of patient movement artifacts on the measurement. Additionally, comparative signal‐to‐noise ratio (SNR) plots are provided for the resting state fMRI, as well as the complete language fMRI, and the intermittent resting state data (see Figure 2B). The data were denoised using Nilearn, applying a high‐pass filter of 0.01 Hz, z‐standardization, and demeaning. CompCor denoising strategy was chosen, selecting anatomical components of the combined white matter and cerebrospinal fluid that explained at least 50% of variance, and the six motion correction parameters and their derivatives (24 parameters). Smoothing was carried out with a Gaussian kernel of 6 mm full‐width at half‐maximum.

FIGURE 2Quality check plots for the resting state and intermittent resting state data. (A) Averaged motion parameter plots over all subjects. (B) Averaged signal‐to‐noise ratio plots, calculated as the mean voxel activation divided by the standard deviation of voxel activation averaged over all subjects. (C) Average time‐series of the default mode network region of interests calculated over all subjects.

**Figure:**
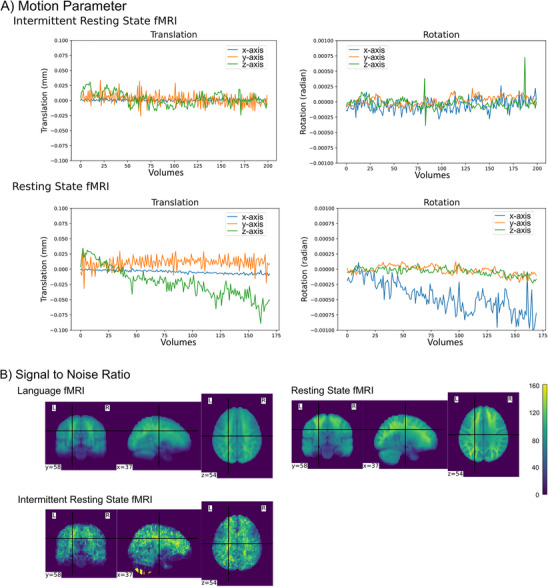

**Figure:**
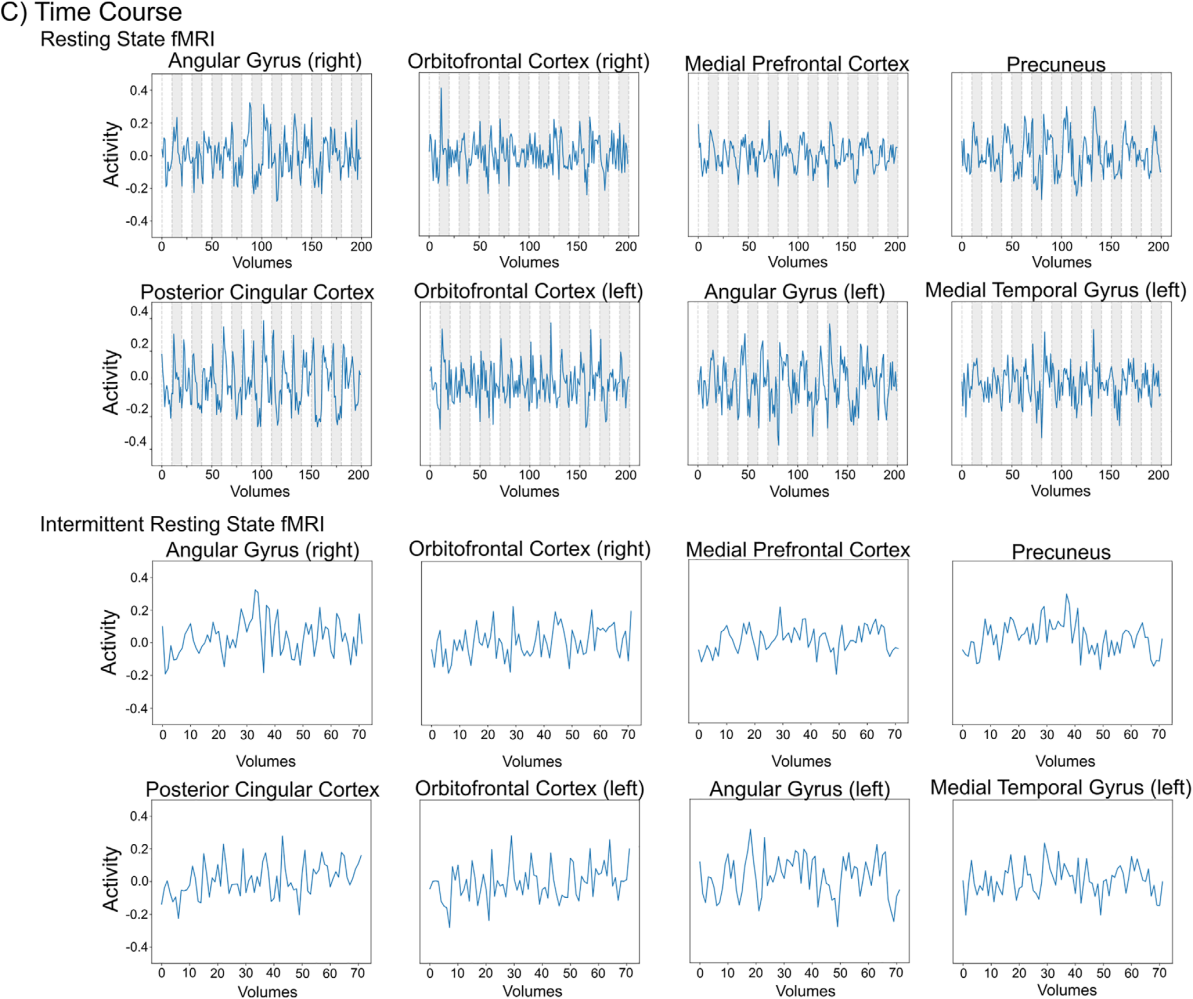

### 2.3 Extracting Rest Data From Language fMRI

Prior to any analysis, rest periods from the language fMRI were extracted according to Fair and colleagues [13]. We chose to use the “interleaved data” approach (as opposed to regressing task from the data) as it produces more similar results in functional connectivity in comparison to continuous resting state data in the study by Fair et al. [13]. The language fMRI is designed with alternating blocks of each 10 rest and task blocks, starting with a rest condition. The number of extracted volumes was adjusted to the repetition time of our data. At the beginning of every scan, three volumes (9 s) were discarded for the signal to reach a baseline level. At the beginning of every task block, two volumes were extracted to be accounted for the “rest” condition. At the beginning of every (except for the very first) rest block, five volumes were discarded for the slow hemodynamic BOLD response of the task to reach baseline level again. After this extraction procedure, out of the 200 volumes acquired in the language fMRI, 72 volumes remained for the intermittent resting state condition of the language fMRI to be further analyzed. Using the same number of volumes for both methods could strengthen the similarity of both methods. Therefore, we additionally subsampled the resting state data by extracting the middle 72 volumes to match the length of the intermittent resting state data.

### 2.4 Statistical Analyses

Statistical analyses were carried out using the CONN Toolbox version 22.a [42, 43] (McGovern Institute for Brain Research, MIT, Cambridge, MA). Preprocessed and denoised data of both methods were analyzed separately using an independent component analysis (ICA), extracting 20 components each, as this number has been shown to efficiently detect resting state networks [44]. Resulting components were compared to the Yeo 7 resting state networks atlas [45] to identify the network that best matches the DMN and the SMN. Resulting correlations of the best matching ICA component of each method with the Yeo DMN and SMN template were tested for differences. To compare within‐network connectivity of both methods, an intersection of networks of both methods was computed and then used as a mask for regions of interest (ROI)‐to‐ROI analyses within each method. Resulting Fisher z‐standardized correlation matrices of both methods were statistically compared using Spearman's rho and nonparametric Monte Carlo permutation testing with 5000 iterations, as well as the Mantel Test for small matrices of the SMN. For group‐level analysis, a seed‐based connectivity analysis using 32 HPC‐ICA network ROIs (CONN Toolbox [43]) with left and right hippocampi as seeds was carried out comparing temporal lobe epilepsy (TLE) patients with left (lTLE) and right (rTLE) hemispheric epileptic focus. This analysis was conducted because previous studies have identified differences in functional connectivity in people with left and right hemisphere epileptic seizure onset focus [46, 47] and highlighted the hippocampi as critical structures [48, 49]. Pereira et al. [50] reported differences when specifically comparing people with lTLE and rTLE in the functional connectivity of left and right hippocampi. In our study, we sought to replicate these findings using both resting state and intermittent resting state methods to determine if results align. For the cluster‐level inference, random field theory parametric statistics were used with a voxel threshold of *p*_uncorrected_ < 0.001 and cluster threshold of *p*_FDRcorrected_ < 0.05 to identify significantly different clusters between the groups of people with lTLE and rTLE.

## 3 Results

### 3.1 Topography of the Default Mode Network

**FIGURE 3:**
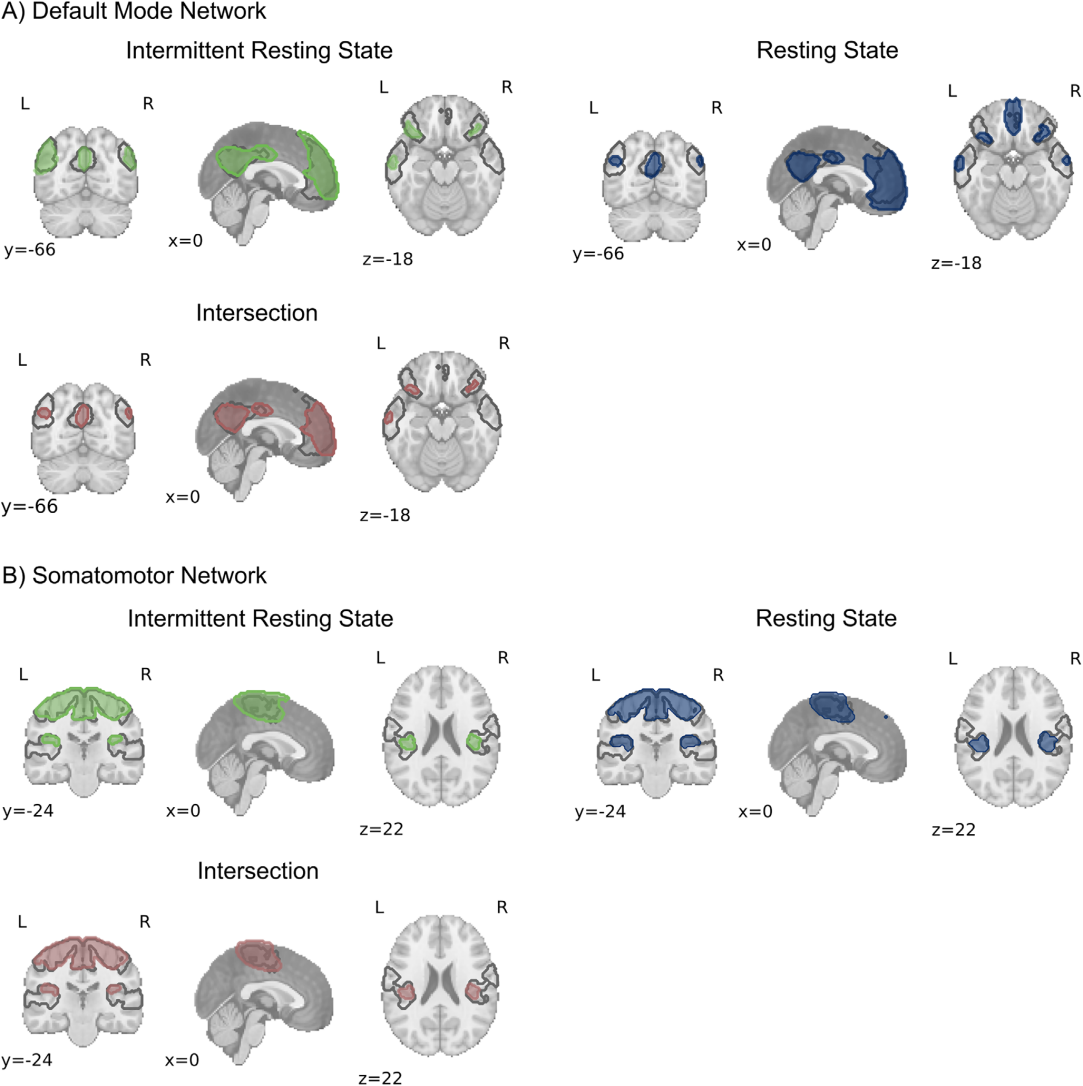
Topography of the independent component analysis components best matching the Yeo Template (given as reference in gray outline). (A) Default mode network. (B) Somatomotor network. Green = component of the intermittent resting state fMRI; Blue = component of the resting state fMRI; Red = intersection of the resting state and intermittent resting state fMRI.

Best matching ICA components of both measures with the Yeo DMN template presented with moderate similarities to the template: *r*_intermittent resting state_ = 0.60, *r*_resting state_ = 0.47. The topography of the subsampled resting state data comprising only 72 volumes was inferior to the complete resting state data missing lateral parietal clusters of the DMN and showing a slightly lower similarity to the Yeo DMN template (*r* = 0.43). Therefore, subsequent analyses were conducted using the complete resting state data. Identified components in relation to the Yeo DMN template can be seen in Figure 3. For the resting state fMRI DMN regions included: orbitofrontal cortex, MPFC, middle temporal gyri (mTG), anterior cingulate cortex, as well as PCC and PCu, as well as small proportions of the angular gyri; all regions were found bilaterally. Similarly, the DMN in the intermittent resting state fMRI comprised the orbitofrontal cortices, anterior cingulate cortex, PCC, PCu, as well as angular gyri in both hemispheres, and the MPFC and mTG in the left hemisphere. Regions from the identified DMN components showed differences, especially in the mTG (missing in the right hemisphere for the intermittent resting state) and angular gyri (resting state showed smaller regions). Both correlated moderately with each other (*r* = 0.51). Correlations did not significantly differ between both acquisition methods (*p* = 0.11), although there was a trend toward higher similarity of the Yeo DMN with the intermittent resting state network. The calculated intersection of both DMNs—only containing regions activated in both DMNs—included eight spatially distinct regions of interest (see Figure 3A): bilateral orbitofrontal cortices, anterior cingulate cortices, PCC, PCu, angular gyri, the MPFC, and mTG in the left hemisphere.

### 3.2 Within‐Network Connectivity of the Default Mode Network

**FIGURE 4:**
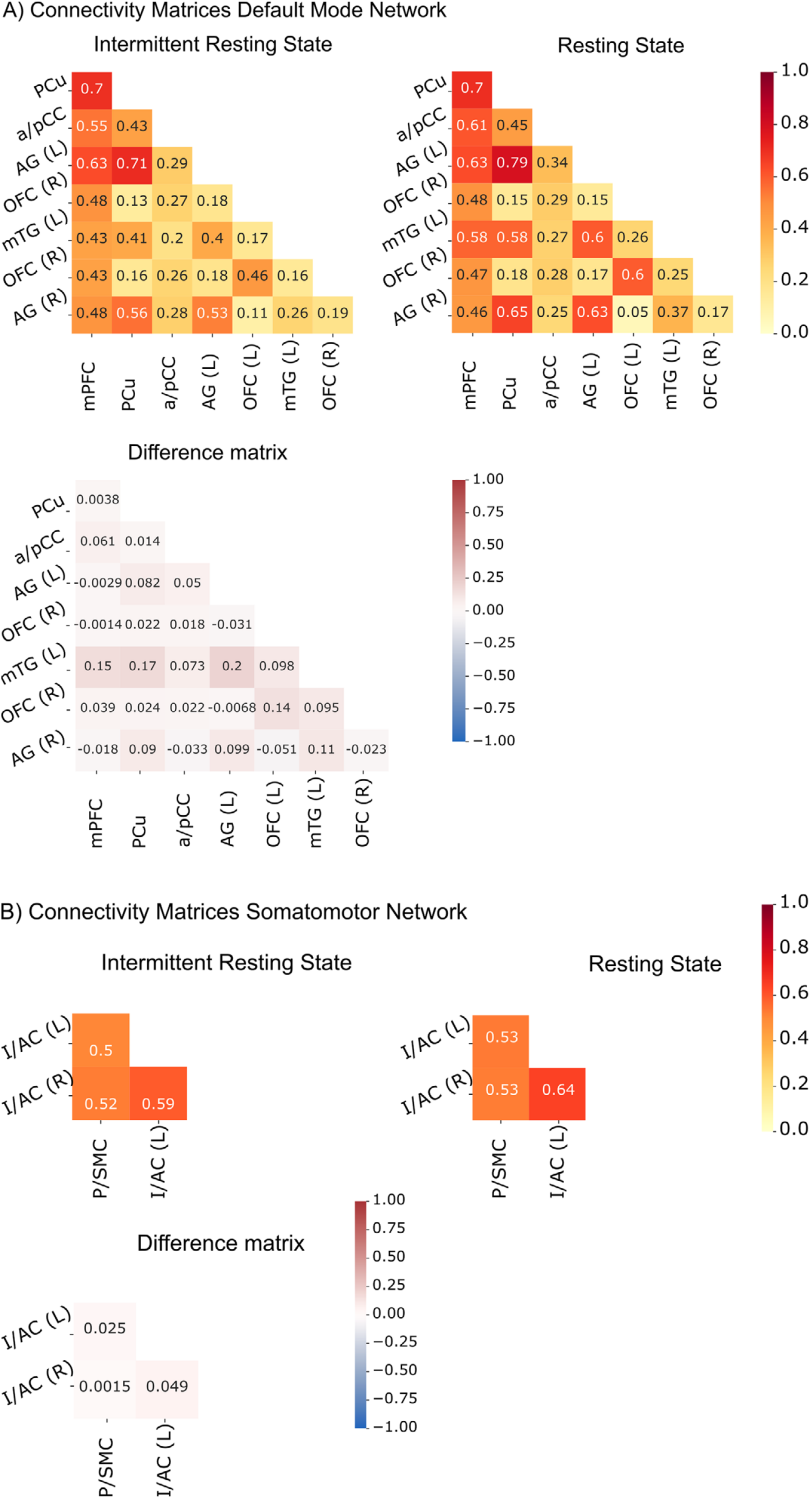
Regions of interest (ROI)‐to‐ROI connectivity matrices for intermittent resting state and resting state data as well as difference matrix (resting state—intermittent resting state). (A) Default mode network connectivity and (B) somatomotor network connectivity. Abbreviations: a/pCC, anterior/posterior cingular cortex; AG, angular gyrus; I/AC, insular‐auditory cortex; L, left; mPFC, medial prefrontal cortex; mTG, medial temporal cortex; OFC, orbitofrontal cortex; PCu, precuneus; P/SMC, primary/supplementary motor cortex; R, right. *Note*: In the difference matrix, red colors and positive values indicate higher connectivity for resting state fMRI.

ROI‐to‐ROI analyses between these eight regions within each fMRI condition yielded similar correlation matrices with highly similar patterns of connectivity (Figure 4A). Similarity analysis showed high similarity in within‐network connectivity of both measures (*r* = 0.93, *p* < 0.01), with the resting state fMRI presenting with slightly higher connectivity (see Figure 4A).

### 3.3 Topography of the Somatomotor Network

For the SMN, similar correlations with the Yeo SMN template were found: *r*_intermittent resting state_ = 0.53, *r*_resting state_ = 0.56. Networks of both methods and the intersection comprise three regions: (1) bilateral primary and supplementary motor cortex; (2) left insular‐auditory cortex; and (3) right insular‐auditory cortex (see Figure 4B).

### 3.4 Within‐Network Connectivity of the Somatomotor Network

ROI‐to‐ROI analyses between the three regions within each fMRI condition yielded similar correlation matrices with highly similar patterns of connectivity (Figure 4B). Similarity analysis showed high similarity in within‐network connectivity of both methods (*r* = 0.97, *p* = 0.33), with the resting state fMRI presenting slightly higher connectivity.

### 3.5 Group‐Level Seed‐Based Connectivity

**FIGURE 5:**
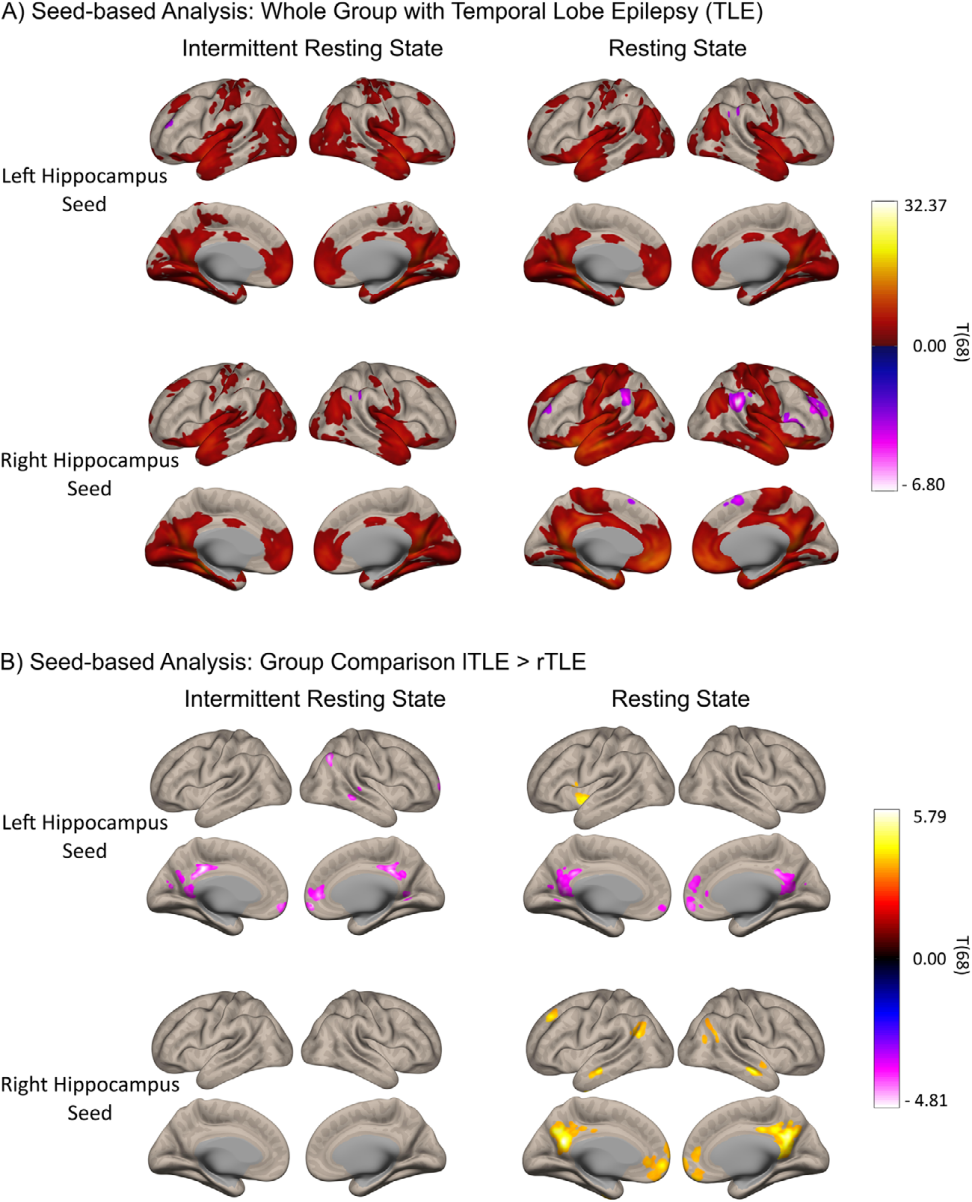
Seed‐based analysis results. (A) Whole group results for people with TLE. Warm colors show positive connectivity, and cool colors show negative connectivity with the hippocampal seed. (B) Results for comparison of people with left TLE (lTLE) and right TLE (rTLE). Warm colors represent higher connectivity for lTLE, cooler colors for rTLE.

Seed‐based analysis for the whole group of people with TLE showed similar connectivity of left and right hippocampus with the rest of the brain for the intermittent and the resting state data, with only small differences (see Figure 5A). Differences were most pronounced for the right hippocampus seed. Here, the intermittent resting state data showed less significant connectivity with the whole brain than the continuous resting state data. To depict the variability between individuals, results of single cases for the comparisons between intermittent and continuous resting state connectivity are displayed in Figure 6. We selected cases with some individuals showing clearer functional connectivity results for the intermittent resting state (ID: 3), some for the continuous resting state (ID: 4), and some similar (ID: 1) or no clear (ID: 2) results for both methods.

FIGURE 6Individual seed‐based functional connectivity results comparing the intermittent and continuous resting state methods. Left and right hippocampus seeds were used.

**Figure:**
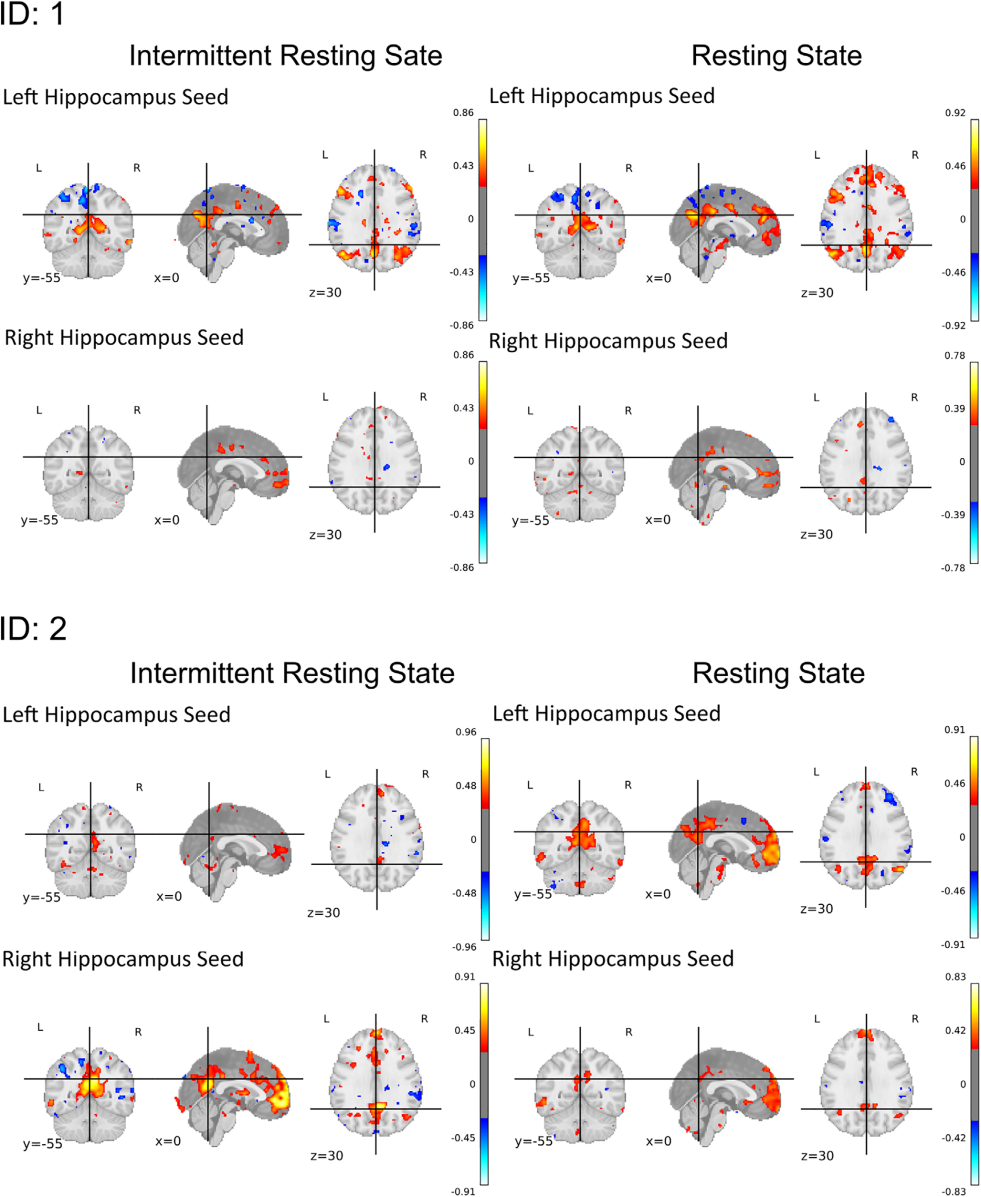

**Figure:**
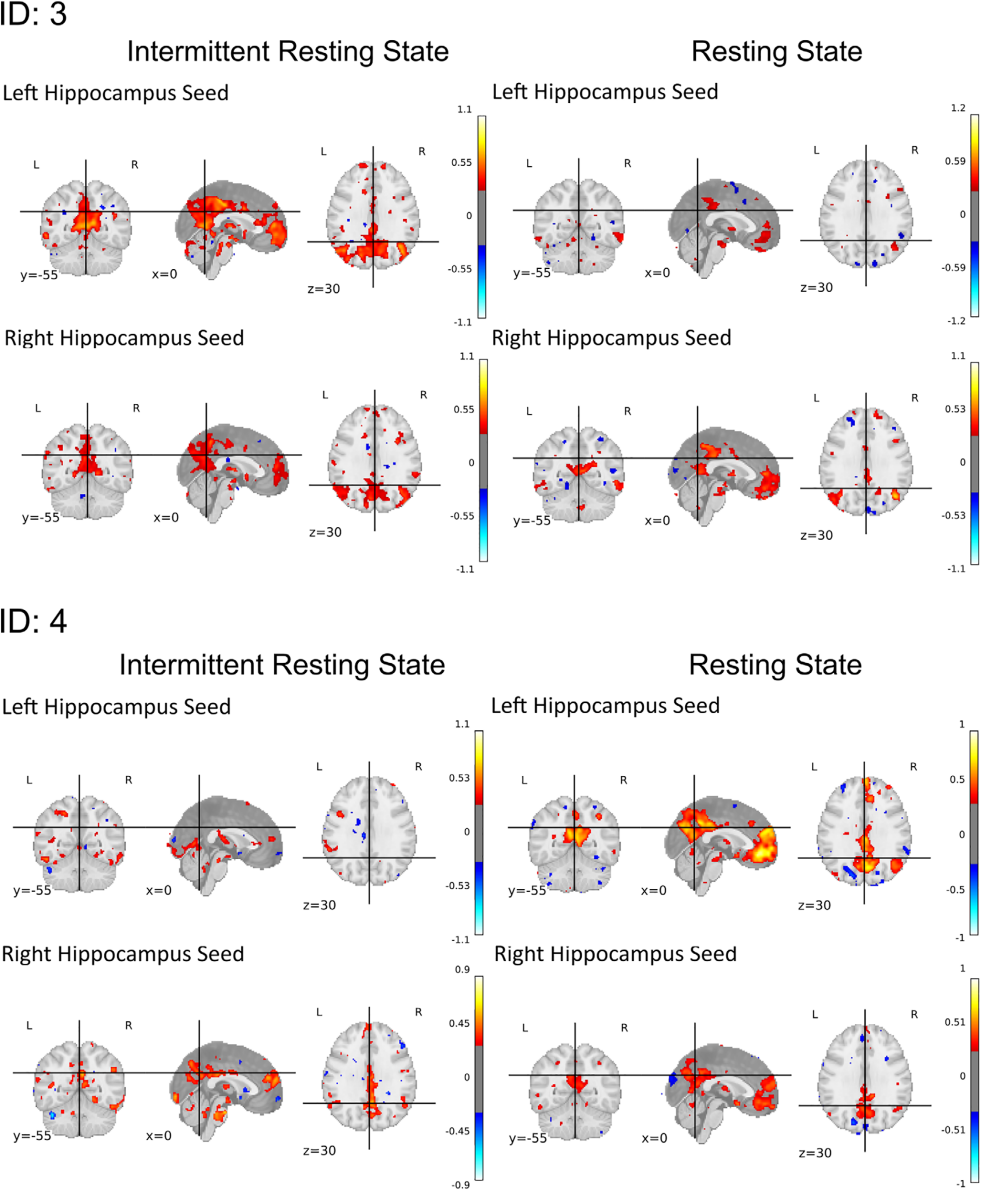

In the group comparison analysis, both methods yielded significant group comparison differences between lTLE and rTLE in the connectivity from the left hippocampal seed to the PCC, the PCu, and the MPFC (see Figure 5B); people with rTLE mostly showed higher connectivity of these regions with the left hippocampus. For the right hippocampal seed, the resting state fMRI showed significant group‐level differences between lTLE and rTLE in the connectivity with the PCC, the PCu, temporal, occipital, and medial frontal cortex regions (see Figure 5B); people with lTLE mostly showed higher connectivity of these regions with the right hippocampus. For the intermittent resting state fMRI, no significant clusters of differences between rTLE and lTLE were found applying the right hippocampal seed (see Figure 5B).

## 4 Discussion

In this study, we investigated whether rest periods from a clinical task‐dependent language fMRI in a blocked design (intermittent resting state) can be used analogously to a continuous resting state fMRI to derive a DMN and SMN in people with epilepsy. Results of topographic analyses, within‐network connectivity, and second‐level group comparison consistently suggest that the intermittent resting state and the resting state are comparable measures of functional connectivity and can be used in a similar manner in people with epilepsy.

### 4.1 Topography of the Resting State Networks

The results demonstrated that a DMN can be derived from both measures, and that there is no significant difference in their topographical correspondence with a reference DMN template [45]. This also applies to the SMN. It aligns with past research in healthy adults showing that task‐dependent fMRI measurements can be used similarly to continuous resting state networks [13, 14, 17].

Specifically, results of topographical similarity with a reference DMN and SMN were comparable to those of a study involving healthy subjects: for both the intermittent resting state method (Beresniewicz et al. [14]: 0.56; our results: 0.60) as well as the resting state method (Beresniewicz et al. [14]: 0.56; our results: 0.47), thus underlining the validity of the applied method in a clinical sample of people with epilepsy.

Regarding differences in the topography of the derived networks, the intermittent resting state fMRI showed pronounced parietal clusters of activation in the angular gyri for the DMN. Previous research has shown that the angular gyrus is associated with mind‐wandering, a state of inward turned attention, and the occurrence of task‐unrelated, self‐generated thought [12, 51]. Mind‐wandering was observed to activate the DMN—and the angular gyrus in particular—in numerous studies [12, 52, 53] as it reflects a “resting” state of the brain in the absence of external stimuli [51]. The strong activation in this particular region in the intermittent resting state fMRI may thus indicate that even the short resting periods between tasks in this design can capture a resting state network of inward‐focused attention. The topography of the SMN was very similar in both the intermittent resting state and the traditional resting state method, and did not show striking differences.

### 4.2 Within‐Network Connectivity of the Resting State Networks

Within‐network connectivity revealed high similarity in connectivity patterns for both networks, which implies that both methods are comparable measures of connectivity and can both be used comparably to map functional connectivity between regions of the DMN and SMN in people with epilepsy. When comparing the magnitude of connectivity between the two measures, functional connectivity was overall slightly higher for the continuous resting state compared to the intermittent resting state (see Figure 4A,B), but not significantly different for the DMN. The biggest difference of connectivity strength was present in ROI‐to‐ROI connectivity of the DMN for the right mTG, with the resting state fMRI showing higher connectivity. Associated with this, results of topographical analysis revealed differences in coactivation within the DMN for the left but not right mTG for the intermittent resting state fMRI as opposed to the resting state fMRI. Given that the mTG is particularly involved in semantic processing [54, 55], which constitutes the primary cognitive demand of the language fMRI task—requiring participants to generate words from a specific category—this observation may result from a subtle influence of task‐related activation on the resting periods. The weaker connectivity with the mTG in the intermittent resting state condition could, therefore, be explained by the downregulation of the task‐activated regions in the following rest period and the DMN [56, 57, 58].

### 4.3 Group‐Level Seed‐Based Analyses

Across people with TLE, hippocampal seed‐based connectivity analysis yielded similar significant effects for the intermittent resting state and the resting state method. Group comparison analyses showed comparable results for the intermittent resting state and the resting state method in patterns of left hippocampus connectivity. In detail, applying the resting state method, people with rTLE exhibited an overall higher connectivity of the PCC and the mPFC to the left hippocampus than lTLE, whereas those with lTLE demonstrated increased connectivity with these regions and the right hippocampus. These results align with previous research demonstrating reduced connectivity of PCC/PCu and mPFC regions with the ipsilateral hippocampus in lTLE and rTLE [59]. Unexpectedly, for the intermittent resting state fMRI for right hippocampal connectivity, no cluster of voxels differed between groups. Whole‐group connectivity in the intermittent resting state generally showed less significant connectivity for the right hippocampal seed to other brain regions. It can be hypothesized that the group differences were, therefore, not large enough to attain statistical significance. In accordance with this, Pereira et al. [50] identified weaker effects and smaller clusters of differences for right hippocampal connectivity when comparing lTLE and rTLE, as opposed to left hippocampal connectivity. Additionally, Fair et al. [13] reported reduced correlation coefficients for their intermittent resting state condition compared to the resting state data. This may also imply that the intermittent resting state method could underestimate certain differences of a small effect size. Despite these minor discrepancies, it can still be argued that the intermittent resting state method is capable of capturing similar group‐level effects as the standard resting state method, albeit with slightly reduced power. It is important to note that some effects might be overlooked with this method, which should be addressed in future research and considered in the application of this method.

### 4.4 Methodological Differences

Regarding the acquisition methods, both fMRI measures show technical differences that could influence the quality of the results. First, the number of analyzed volumes differs between methods: 170 volumes for the resting state and 72 volumes for the intermittent resting state fMRI. Previous studies applying a similar technique included a comparable—or even smaller—amount of volumes in their analyses, still yielding good results also in comparison to a continuous resting state scan [14, 15, 16]. When using the same number of volumes for both methods, we observed poorer results for the shorter resting state data (i.e., missing lateral parietal clusters of the DMN template). By using the complete resting state data, we furthermore best reflect the effective comparison with a standard continuous resting state fMRI in clinical settings. Additionally, results already suggest high similarity regardless of the differing number of volumes. Although the intermittent resting state does not consist of a continuous measure of brain signal, it can be argued that the ongoing switch of task activation and resting periods may significantly reduce the risk for a drift toward drowsiness or even sleep, which is known to impair the quality of a resting state measurement [60, 61, 62]. Taken together, it can be asserted that despite fewer time points and duration of analyzed scans, the intermittent resting state fMRI is still a valid measure of functional connectivity and even presents with some advantages over the resting state fMRI.

A further difference between our language fMRI and the resting state fMRI lies in different acquisition parameters and receive coils. As shown in the temporal SNR plots (Figure 2B), the resting state acquisition yielded higher SNR, whereas the SNR was lower for the intermittent resting state method. These discrepancies may affect the sensitivity and robustness of the connectivity analyses. However, the substantial similarity of the connectivity results obtained with both methods suggests that the main conclusions of the study remain valid despite these differences.

Another difference is the instruction given prior to the scan. For the continuous resting state measurement, participants were instructed to keep their eyes open and think of nothing in particular. Before the language fMRI, participants were advised to start performing the task and stop as soon as they were given the cue. Thus, the overall focus was rather set on the task than on the rest periods. One could argue that there was an influence of the task to the following rest block and, therefore, the rest period is not as clear and free of task influence. This was somewhat reflected in the spatial architecture and in the connectivity patterns. On the contrary, the topography of the DMN was slightly better fitting (in comparison to a standard DMN template) than the resting state DMN. This accords with Pyka et al. [56], who found that after a cognitively demanding task, the DMN activation was higher. Riemer et al. [58] explained this by the upregulation of the DMN when switching from an active to a passive (rest) state.

Beresniewicz et al. [14] recommended exclusion of volumes from the end of the rest block and beginning of the task block to avoid interfering with the anticipatory processes of the task. Such influences were shown for cognitively demanding tasks (such as the n‐back task) [56]. Because of the clinical routine nature of the data, no variation of task difficulty and cognitive load could be examined. But since analysis of DMN ROI‐based time‐series did not show systematic activation changes at the end of the rest block (cf. end segments of white blocks in Figure 2C), an anticipatory effect is less likely to impact the data presented in our study. Beresciewicz et al. [14] additionally argued that differences between intermittent and continuous resting state methods could be attributed to “carry‐over” effects of the task to the rest period. This would lead to reduced self‐referential processes and mind‐wandering and—as a result—to reduced MPFC activity in the intermittent resting state data. Notably, such effects could not be observed in our study: intermittent and continuous resting state data showed comparably pronounced activation in the MPFC (see Figure 3), arguing against the existence of marked carry‐over effects in our intermittent resting state data. Nevertheless, as we have not specifically investigated possible carry‐over effects, an influence cannot be precluded with certainty. In sum, the use of our method with other tasks could impose the potential risk of confounding the rest blocks of the intermittent resting state by anticipation of the task or “carry‐over” effects to the rest periods [14]. Future application of the method using other tasks should thus include a systematic use of postscan questionnaires, careful inspection of activity changes for the last volumes of the rest periods, and of MPFC activity.

### 4.5 Limitations

In this study, we chose to focus on DMN functional connectivity due to the relevance of the DMN for clinical applications in people with epilepsy [26, 30, 31, 32]. As a strength of our study, we additionally included results from SMN connectivity to obtain a more comprehensive perspective. However, more detailed analyses of other resting state networks and in additional clinical cohorts should be conducted. Further, it would be interesting to analyze the task periods of the language fMRI, as studies suggest that functional resting state connectivity can also be derived from a mere task‐dependent state [63].

Based on the most similar results for the “interleaved” method compared to a continuous resting state in the study by Fair et al. [13], we chose this method to apply in our study. However, it remains unclear whether other approaches (such as deconvolving [13, 64]) would also be effective. These comparisons should be investigated in future studies.

Additionally, a reference to a healthy control group may have been useful for evaluating the similarity results found in our study. Still, we underline that our similarity of the DMN topography to a reference DMN yielded comparable results as previous studies in healthy controls [14], as discussed above. Comparing the topographical similarity of both methods, previous studies reported slightly higher similarities between both methods in a healthy sample (Zhu et al. [17]: 0.78/0.71, our results: 0.51). However, due to the different analysis approach (i.e., ICA in our study vs. seed‐based connectivity in Zhu et al. [17]), this comparison should be drawn with caution. Future studies should, therefore, employ such between‐method comparisons in healthy controls using our ICA approach, which would eventually help evaluating the specificity of our similarity results in a clinical cohort of people with epilepsy.

### 4.6 Conclusion and Implications

With this study, we aimed to extend the validation of the intermittent resting state method to a clinical sample. This was accomplished in a cohort of people with epilepsy, for whom task‐dependent fMRI is routinely performed in clinical practice. By demonstrating for the first time that the intermittent resting state method is suitable for functional connectivity analysis of the DMN in people with epilepsy, we facilitate its easier application in clinical practice, eliminating the need for an additional resting state measurement. Hence, the intermittent resting state method produces similar results to a resting state method in a group of people who exhibit alterations in functional connectivity and may even offer certain advantages over a continuous resting state measurement.

Thus, the intermittent resting state method could provide an excellent cost‐ and time‐efficient opportunity to derive a DMN from a task‐dependent clinical fMRI without additionally performing a resting state fMRI. This capability allows it to be used not only for research purposes but also in clinical practice. The implementation of this method can extend the understanding of functional brain network functionality, given the relevance of epilepsy research in the context of neuroscience [65]. It can also be employed to investigate cognitive impairment, such as memory deficits. Yet, future studies are needed to investigate whether intermittent resting state data, as generated in our study, may also be used in further application fields, such as using resting state data to train artificial intelligence models to localize epileptic seizures [66]. Still, the intermittent resting state method presented in our study holds potential for a variety of possible applications in the field of clinical neuroscience and epilepsy research.

## Funding

This work was supported by the Deutsche Forschungsgemeinschaft [grant numbers LA 3567/2‐1, BI 1254/9‐1]. PG holds the Friedrich von Bodelschwingh endowed Junior‐Professorship for “Clinical Neuropsychology and Epilepsy Research” at the Medical School OWL, Bielefeld University, funded by the v. Bodelschwingh Foundation.

## Conflicts of Interest

None of the authors has any conflicts of interest to disclose. The funding sources did not have any influence on the study's design, data collection, analyses, interpretation, manuscript preparation, and submission.

This manuscript has been made available as a preprint on PsyArXiv, with the DOI: 10.31234/osf.io/6uvs9_v1 on May 12, 2025.

## References

[1] J. S. Damoiseaux and M. D. Greicius , “Greater Than the Sum of Its Parts: A Review of Studies Combining Structural Connectivity and Resting‐State Functional Connectivity,” Brain Structure and Function 213 (2009): 525–533. 19565262 10.1007/s00429-009-0208-6

[2] K. A. Smitha , K. Akhil Raja , and K. M. Arun , “Resting State fMRI: A Review on Methods in Resting State Connectivity Analysis and Resting State Networks,” Neuroradiology Journal 30 (2017): 305–317. 28353416 10.1177/1971400917697342 PMC5524274

[3] M. P. van den Heuvel and H. E. Hulshoff Pol , “Exploring the Brain Network: A Review on Resting‐State fMRI Functional Connectivity,” European Neuropsychopharmacology 20 (2010): 519–534. 20471808 10.1016/j.euroneuro.2010.03.008

[4] K. J. Friston , C. D. Frith , P. F. Liddle , and R. S. Frackowiak , “Functional Connectivity: The Principal‐Component Analysis of Large (PET) Data Sets,” Journal of Cerebral Blood Flow and Metabolism 13 (1993): 5–14. 8417010 10.1038/jcbfm.1993.4

[5] C. F. Beckmann , M. DeLuca , J. T. Devlin , and S. M. Smith , “Investigations Into Resting‐State Connectivity Using Independent Component Analysis,” Philosophical Transactions of the Royal Society of London. Series B: Biological Sciences 360 (2005): 1001–1013. 16087444 10.1098/rstb.2005.1634 PMC1854918

[6] J. S. Damoiseaux , S. Rombouts , F. Barkhof , et al., “Consistent Resting‐State Networks Across Healthy Subjects,” Proceedings of the National Academy of Sciences 103 (2006): 13848–13853. 10.1073/pnas.0601417103 PMC1564249 16945915

[7] M. E. Raichle , A. M. MacLeod , A. Z. Snyder , W. J. Powers , D. A. Gusnard , and G. L. Shulman , “A Default Mode of Brain Function,” Proceedings of the National Academy of Sciences 98 (2001): 676–682. 10.1073/pnas.98.2.676 PMC14647 11209064

[8] M. D. Greicius , B. Krasnow , A. L. Reiss , and V. Menon , “Functional Connectivity in the Resting Brain: A Network Analysis of the Default Mode Hypothesis,” Proceedings of the National Academy of Sciences 100 (2003): 253–258. 10.1073/pnas.0135058100 PMC140943 12506194

[9] R. L. Buckner , J. R. Andrews‐Hanna , and D. L. Schacter , “The Brain's Default Network: Anatomy, Function, and Relevance to Disease,” Annals of the New York Academy of Sciences 1124 (2008): 1–38. 18400922 10.1196/annals.1440.011

[10] M. E. Raichle , “The Brain's Default Mode Network,” Annual Review of Neuroscience 38 (2015): 433–447. 10.1146/annurev-neuro-071013-014030 25938726

[11] M. D. Fox , A. Z. Snyder , J. L. Vincent , M. Corbetta , D. C. van Essen , and M. E. Raichle , “The Human Brain Is Intrinsically Organized Into Dynamic, Anticorrelated Functional Networks,” Proceedings of the National Academy of Sciences 102 (2005): 9673–9678. 10.1073/pnas.0504136102 PMC1157105 15976020

[12] C. L. Philippi , J. Bruss , A. D. Boes , et al., “Lesion Network Mapping Demonstrates That Mind‐Wandering Is Associated With the Default Mode Network,” Journal of Neuroscience Research 99 (2021): 361–373. 32594566 10.1002/jnr.24648 PMC7704688

[13] D. A. Fair , B. L. Schlaggar , A. L. Cohen , et al., “A Method for Using Blocked and Event‐Related fMRI Data to Study “Resting State” Functional Connectivity,” Neuroimage 35 (2007): 396–405. 17239622 10.1016/j.neuroimage.2006.11.051 PMC2563954

[14] J. Beresniewicz , F. Riemer , K. Kazimierczak , et al., “Similarities and Differences Between Intermittent and Continuous Resting‐State fMRI,” Frontiers in Human Neuroscience 17 (2023): 1238888. 37600552 10.3389/fnhum.2023.1238888 PMC10435290

[15] D. Pareto , J. Sastre‐Garriga , J. Alonso , et al., “Classic Block Design “Pseudo”‐Resting‐State fMRI Changes After a Neurorehabilitation Program in Patients With Multiple Sclerosis,” Journal of Neuroimaging 28 (2018): 313–319. 29400912 10.1111/jon.12500

[16] M. Loitfelder , M. Filippi , M. Rocca , et al., “Abnormalities of Resting State Functional Connectivity Are Related to Sustained Attention Deficits in MS,” PLoS ONE 7 (2012): e42862. 22912754 10.1371/journal.pone.0042862 PMC3422320

[17] Y. Zhu , L. Cheng , N. He , et al., “Comparison of Functional Connectivity Estimated From Concatenated Task‐State Data From Block‐Design Paradigm With That of Continuous Task,” Computational and Mathematical Methods in Medicine 2017 (2017): 4198430. 28191030 10.1155/2017/4198430 PMC5278200

[18] M. Cataldi , M. Avoli , and V.‐S. E. de , “Resting State Networks in Temporal Lobe Epilepsy,” Epilepsia 54 (2013): 2048–2059. 24117098 10.1111/epi.12400 PMC4880458

[19] H. Burianová , N. L. Faizo , M. Gray , J. Hocking , G. Galloway , and D. Reutens , “Altered Functional Connectivity in Mesial Temporal Lobe Epilepsy,” Epilepsy Research 137 (2017): 45–52. 28923408 10.1016/j.eplepsyres.2017.09.001

[20] W. Liao , Z. Zhang , Z. Pan , et al., “Default Mode Network Abnormalities in Mesial Temporal Lobe Epilepsy: A Study Combining fMRI and DTI,” Human Brain Mapping 32 (2011): 883–895. 20533558 10.1002/hbm.21076 PMC6870458

[21] F. Pittau , C. Grova , F. Moeller , F. Dubeau , and J. Gotman , “Patterns of Altered Functional Connectivity in Mesial Temporal Lobe Epilepsy,” Epilepsia 53 (2012): 1013–1023. 22578020 10.1111/j.1528-1167.2012.03464.x PMC3767602

[22] V. R. Desai , A. Vedantam , S. K. Lam , et al., “Language Lateralization With Resting‐State and Task‐Based Functional MRI in Pediatric Epilepsy,” Journal of Neurosurgery: Pediatrics 23 (2019): 171–177. 30485177 10.3171/2018.7.PEDS18162

[23] G. E. Doucet , D. Pustina , C. Skidmore , A. Sharan , M. R. Sperling , and J. I. Tracy , “Resting‐State Functional Connectivity Predicts the Strength of Hemispheric Lateralization for Language Processing in Temporal Lobe Epilepsy and Normals,” Human Brain Mapping 36 (2015): 288–303. 25187327 10.1002/hbm.22628 PMC4295620

[24] S. M. Stufflebeam , H. Liu , J. Sepulcre , N. Tanaka , R. L. Buckner , and J. R. Madsen , “Localization of Focal Epileptic Discharges Using Functional Connectivity Magnetic Resonance Imaging,” Journal of Neurosurgery 114 (2011): 1693–1697. 21351832 10.3171/2011.1.JNS10482 PMC3248962

[25] Z. Y.‐F. Wurina and S.‐G. Zhao , “Resting‐State fMRI Studies in Epilepsy,” Neuroscience Bulletin 28 (2012): 449–455. 22833042 10.1007/s12264-012-1255-1 PMC5561896

[26] C. McCormick , M. Quraan , M. Cohn , T. A. Valiante , and M. P. McAndrews , “Default Mode Network Connectivity Indicates Episodic Memory Capacity in Mesial Temporal Lobe Epilepsy,” Epilepsia 54 (2013): 809–818. 23360362 10.1111/epi.12098

[27] G. E. Doucet , R. Rider , N. Taylor , et al., “Presurgery Resting‐State Local Graph‐Theory Measures Predict Neurocognitive Outcomes After Brain Surgery in Temporal Lobe Epilepsy,” Epilepsia 56 (2015): 517–526. 25708625 10.1111/epi.12936

[28] R. Garner , M. La Rocca , G. Barisano , A. W. Toga , D. Duncan , and P. Vespa , “A Machine Learning Model to Predict Seizure Susceptibility From Resting‐State fMRI Connectivity,” Spring Simulation Conference 2019 (2019). 10.23919/springsim.2019.8732859 PMC9760283 36541915

[29] M. Negishi , R. Martuzzi , E. J. Novotny , D. D. Spencer , and R. T. Constable , “Functional MRI Connectivity as a Predictor of the Surgical Outcome of Epilepsy,” Epilepsia 52 (2011): 1733–1740. 21801165 10.1111/j.1528-1167.2011.03191.x PMC3169719

[30] S. Jiang , H. Li , L. Liu , D. Yao , and C. Luo , “Voxel‐Wise Functional Connectivity of the Default Mode Network in Epilepsies: A Systematic Review and Meta‐Analysis,” Current Neuropharmacology 20 (2022): 254–266. 33823767 10.2174/1570159X19666210325130624 PMC9199542

[31] A. Barnett , S. Audrain , and M. P. McAndrews , “Applications of Resting‐State Functional MR Imaging to Epilepsy,” Neuroimaging Clinics of North America 27 (2017): 697–708. 28985938 10.1016/j.nic.2017.06.002

[32] A. Doll , M. Wegrzyn , A. Benzait , et al., “Whole‐Brain Functional Correlates of Memory Formation in Mesial Temporal Lobe Epilepsy,” NeuroImage: Clinical 31 (2021): 102723. 34147817 10.1016/j.nicl.2021.102723 PMC8220377

[33] D. Li , R. Liu , L. Meng , et al., “Abnormal Ventral Somatomotor Network Homogeneity in Patients With Temporal Lobe Epilepsy,” Frontiers in Psychiatry 13 (2022): 877956. 35782421 10.3389/fpsyt.2022.877956 PMC9247252

[34] M. Carboni , S. P. de , B. J. Vorderwülbecke , et al., “Abnormal Directed Connectivity of Resting State Networks in Focal Epilepsy,” NeuroImage: Clinical 27 (2020): 102336. 32679553 10.1016/j.nicl.2020.102336 PMC7363703

[35] V. L. Vogt , M. Äikiä , A. Del Barrio , et al., “Current Standards of Neuropsychological Assessment in Epilepsy Surgery Centers Across Europe,” Epilepsia 58 (2017): 343–355. 28067423 10.1111/epi.13646

[36] F. G. Woermann , H. Jokeit , R. Luerding , et al., “Language Lateralization by Wada Test and fMRI in 100 Patients With Epilepsy,” Neurology 61 (2003): 699–701. 12963768 10.1212/01.wnl.0000078815.03224.57

[37] A. Doll , M. Wegrzyn , F. G. Woermann , K. Labudda , C. G. Bien , and J. Kissler , “MRI Evidence for Material‐Specific Encoding Deficits and Mesial‐Temporal Alterations in Presurgical Frontal Lobe Epilepsy Patients,” Epilepsia Open 9 (2024): 355–367. 38093701 10.1002/epi4.12881 PMC10839294

[38] A. Doll , D. A. Schlueter , M. Wegrzyn , et al., “Encoding‐Related Hippocampus Connectivity for Scenes, Faces, and Words: Healthy People Compared to People With Temporal and Frontal Lobe Epilepsy,” NeuroImage: Clinical 46 (2025): 103784. 40253948 10.1016/j.nicl.2025.103784 PMC12023899

[39] O. Esteban , C. J. Markiewicz , R. W. Blair , et al., “fMRIPrep: A Robust Preprocessing Pipeline for Functional MRI,” Nature Methods 16 (2019): 111–116. 30532080 10.1038/s41592-018-0235-4 PMC6319393

[40] A. Abraham , F. Pedregosa , M. Eickenberg , et al., “Machine Learning for Neuroimaging With Scikit‐Learn,” Frontiers in Neuroinformatics 8 (2014): 14. 24600388 10.3389/fninf.2014.00014 PMC3930868

[41] Y. Behzadi , K. Restom , J. Liau , and T. T. Liu , “A Component Based Noise Correction Method (CompCor) for BOLD and Perfusion Based fMRI,” Neuroimage 37 (2007): 90–101. 17560126 10.1016/j.neuroimage.2007.04.042 PMC2214855

[42] S. Whitfield‐Gabrieli and A. Nieto‐Castanon , “Conn: A Functional Connectivity Toolbox for Correlated and Anticorrelated Brain Networks,” Brain Connectivity 2 (2012): 125–141. 22642651 10.1089/brain.2012.0073

[43] A. Nieto‐Castanon and S. Whitfield‐Gabrieli , CONN Functional Connectivity Toolbox: RRID SCR_009550, Release 22 (Hilbert Press, 2022).

[44] S. M. Smith , P. T. Fox , K. L. Miller , et al., “Correspondence of the Brain's Functional Architecture During Activation and Rest,” Proceedings of the National Academy of Sciences 106 (2009): 13040–13045. 10.1073/pnas.0905267106 PMC2722273 19620724

[45] B. T. T. Yeo , F. M. Krienen , J. Sepulcre , et al., “The Organization of the Human Cerebral Cortex Estimated by Intrinsic Functional Connectivity,” Journal of Neurophysiology 106 (2011): 1125–1165. 21653723 10.1152/jn.00338.2011 PMC3174820

[46] T. A. Zanão , T. M. Lopes , C. B. M. de , C. L. Yasuda , and F. Cendes , “Patterns of Default Mode Network in Temporal Lobe Epilepsy With and Without Hippocampal Sclerosis,” Epilepsy & Behavior 121 (2021): 106523. 31645315 10.1016/j.yebeh.2019.106523

[47] S. Amiri , J. Mehvari‐Habibabadi , N. Mohammadi‐Mobarakeh , et al., “Graph Theory Application With Functional Connectivity to Distinguish Left From Right Temporal Lobe Epilepsy,” Epilepsy Research 167 (2020): 106449. 32937221 10.1016/j.eplepsyres.2020.106449

[48] S. Chiang , J. M. Stern , J. Engel , H. S. Levin , and Z. Haneef , “Differences in Graph Theory Functional Connectivity in Left and Right Temporal Lobe Epilepsy,” Epilepsy Research 108 (2014): 1770–1781. 25445238 10.1016/j.eplepsyres.2014.09.023 PMC5013648

[49] S. Narasimhan , H. F. J. González , G. W. Johnson , et al., “Functional Connectivity Between Mesial Temporal and Default Mode Structures May Help Lateralize Surgical Temporal Lobe Epilepsy,” Journal of Neurosurgery 137 (2022): 1571–1581. 35364587 10.3171/2022.1.JNS212031 PMC9525455

[50] F. R. S. Pereira , A. Alessio , M. S. Sercheli , et al., “Asymmetrical Hippocampal Connectivity in Mesial Temporal Lobe Epilepsy: Evidence From Resting State fMRI,” BMC Neuroscience [Electronic Resource] 11 (2010): 66. 20525202 10.1186/1471-2202-11-66 PMC2890013

[51] M. L. Seghier , “The Angular Gyrus: Multiple Functions and Multiple Subdivisions,” Neuroscientist 19 (2013): 43–61. 22547530 10.1177/1073858412440596 PMC4107834

[52] K. Christoff , A. M. Gordon , J. Smallwood , R. Smith , and J. W. Schooler , “Experience Sampling During fMRI Reveals Default Network and Executive System Contributions to Mind Wandering,” Proceedings of the National Academy of Sciences 106 (2009): 8719–8724. 10.1073/pnas.0900234106 PMC2689035 19433790

[53] J. Smallwood , K. Brown , B. Baird , and J. W. Schooler , “Cooperation Between the Default Mode Network and the Frontal‐Parietal Network in the Production of an Internal Train of Thought,” Brain Research 1428 (2012): 60–70. 21466793 10.1016/j.brainres.2011.03.072

[54] J. Davey , H. E. Thompson , G. Hallam , et al., “Exploring the Role of the Posterior Middle Temporal Gyrus in Semantic Cognition: Integration of Anterior Temporal Lobe With Executive Processes,” Neuroimage 137 (2016): 165–177. 27236083 10.1016/j.neuroimage.2016.05.051 PMC4927261

[55] L. Papeo , B. Agostini , and A. Lingnau , “The Large‐Scale Organization of Gestures and Words in the Middle Temporal Gyrus,” Journal of Neuroscience 39 (2019): 5966–5974. 31126999 10.1523/JNEUROSCI.2668-18.2019 PMC6650980

[56] M. Pyka , C. F. Beckmann , S. Schöning , et al., “Impact of Working Memory Load on fMRI Resting State Pattern in Subsequent Resting Phases,” PLoS ONE 4 (2009): e7198. 19779619 10.1371/journal.pone.0007198 PMC2745698

[57] K. Hugdahl , K. Kazimierczak , J. Beresniewicz , et al., “Dynamic Up‐ and Down‐Regulation of the Default (DMN) and Extrinsic (EMN) Mode Networks During Alternating Task‐On and Task‐Off Periods,” PLoS ONE 14 (2019): e0218358. 31536496 10.1371/journal.pone.0218358 PMC6752853

[58] F. Riemer , R. Grüner , J. Beresniewicz , K. Kazimierczak , L. Ersland , and K. Hugdahl , “Dynamic Switching Between Intrinsic and Extrinsic Mode Networks as Demands Change From Passive to Active Processing,” Scientific Reports 10 (2020): 21463. 33293637 10.1038/s41598-020-78579-6 PMC7722921

[59] Z. Haneef , A. Lenartowicz , H. J. Yeh , J. Engel , and J. M. Stern , “Effect of Lateralized Temporal Lobe Epilepsy on the Default Mode Network,” Epilepsy & Behavior 25 (2012): 350–357. 23103309 10.1016/j.yebeh.2012.07.019 PMC4209897

[60] E. Tagliazucchi and H. Laufs , “Decoding Wakefulness Levels From Typical fMRI Resting‐State Data Reveals Reliable Drifts Between Wakefulness and Sleep,” Neuron 82 (2014): 695–708. 24811386 10.1016/j.neuron.2014.03.020

[61] J. Wang , J. Han , V. T. Nguyen , L. Guo , and C. C. Guo , “Improving the Test‐Retest Reliability of Resting State fMRI by Removing the Impact of Sleep,” Frontiers in Neuroscience 11 (2017): 249. 28533739 10.3389/fnins.2017.00249 PMC5420587

[62] A. M. Soehner , H. W. Chase , M. A. Bertocci , et al., “Unstable Wakefulness During Resting‐State fMRI and Its Associations With Network Connectivity and Affective Psychopathology in Young Adults,” Journal of Affective Disorders 258 (2019): 125–132. 31401540 10.1016/j.jad.2019.07.066 PMC6710159

[63] F. M. Krienen , B. T. T. Yeo , and R. L. Buckner , “Reconfigurable Task‐Dependent Functional Coupling Modes Cluster Around a Core Functional Architecture,” Philosophical Transactions of the Royal Society of London. Series B: Biological Sciences 369, no. 1653 (2014): 20130526. 25180304 10.1098/rstb.2013.0526 PMC4150301

[64] R. J. Harris , S. Y. Bookheimer , T. F. Cloughesy , et al., “Altered Functional Connectivity of the Default Mode Network in Diffuse Gliomas Measured With Pseudo‐Resting State fMRI,” Journal of Neuro‐Oncology 116 (2014): 373–379. 24234804 10.1007/s11060-013-1304-2 PMC6763342

[65] H. Beck and C. E. Elger , “Epilepsy Research: A Window Onto Function to and Dysfunction of the Human Brain,” Dialogues in Clinical Neuroscience 10 (2008): 7–15. 18472480 10.31887/DCNS.2008.10.1/hbeck PMC3181865

[66] P. H. Luckett , L. Maccotta , J. J. Lee , et al., “Deep Learning Resting State Functional Magnetic Resonance Imaging Lateralization of Temporal Lobe Epilepsy,” Epilepsia 63 (2022): 1542–1552. 35320587 10.1111/epi.17233 PMC9177812

